# Cardiovascular Protective Effects of Plant Polysaccharides: A Review

**DOI:** 10.3389/fphar.2021.783641

**Published:** 2021-11-18

**Authors:** Xinli Dong, Mengze Zhou, Yehong Li, Yuxin Li, Hui Ji, Qinghua Hu

**Affiliations:** ^1^ State Key Laboratory of Natural Medicines, China Pharmaceutical University, Nanjing, China; ^2^ School of Pharmacy, China Pharmaceutical University, Nanjing, China

**Keywords:** plant polysaccharides, cardiovascular diseases, pharmacological effects, protective mechanisms, low side effects

## Abstract

Cardiovascular disease is a kind of heart, brain, and blood vessel injury disease by the interaction of various pathological factors. The pathogenesis of cardiovascular disease is complex with various risk factors, including abnormally elevated blood pressure, glucose, and lipid metabolism disorders, atherosclerosis, thrombosis, etc. Plant polysaccharides are a special class of natural products derived from plant resources, which have the characteristics of wide sources, diverse biological activities, and low toxicity or side effects. Many studies have shown that plant polysaccharides improve cardiovascular diseases through various mechanisms such as anti-oxidative stress, restoring the metabolism of biological macromolecules, regulating the apoptosis cascade to reduce cell apoptosis, and inhibiting inflammatory signal pathways to alleviate inflammation. This article reviews the pharmacological effects and protective mechanisms of some plant polysaccharides in modulating the cardiovascular system, which is beneficial for developing more effective drugs with low side effects for management of cardiovascular diseases.

## Introduction

Cardiovascular disease (CVD) is a type of chronic non-infectious disease caused by circulatory system damage (Abe et al., 2017), with the characteristics of high incidence and large mortality. In recent years, owing to human lifestyle changes, the prevalence of CVD has been on an upward trend, and its fatality rate far exceeds that of cancer and other diseases. Statistically, more than two-fifths of deaths are attributed to CVD, in the rural and urban Chinese death population in 2016 (Ma et al., 2020), which makes CVD become the number one killer that affects human health. With the increasing understanding of the pathogenesis of CVD, the level of medical care of CVD has made great progress. Still, there are some shortcomings in the clinical treatment of CVD that remain to be resolved. In terms of drugs, most of small molecule chemicals commonly used in clinical treatment of CVD have many adverse reactions, insignificant efficacy, low patient compliance and other disadvantages. Consequently, in the process of seeking new drugs, plant polysaccharides with multiple targets, good biocompatibility and low toxicity have gradually become a hot spot in the research of anti-CVD drugs.

As a kind of natural macromolecule polymer extracted from various parts of plants, plant polysaccharides are composed of ten or more monosaccharides through polymerization with glycosidic linkages (Yu et al., 2018). A large number of studies have shown that plant polysaccharides have various bioactivities such as anti-tumor, immunomodulation, antioxidant, radioprotection, hepatoprotection, anti-virus (Xie et al., 2016; Yu et al., 2018), which play an important role in regulating human physiological functions. More importantly, several studies have also shown other functions of plant polysaccharides such as antioxidant, anti-hyperglycemic, anti-hypertensive, anti-atherosclerosis, anti-myocardial ischemia etc. (Zaporozhets and Besednova, 2016). These pharmacological effects provide a theoretical basis for plant polysaccharides to treat CVD. This article reviews reported mechanisms by which plant polysaccharides protect CVD from the perspective of multiple pharmacological effects.

## Protective Effects of Plant Polysaccharides on Cardiovascular System

Globally, CVD is not only the leading cause of the decline in people’s quality of life, but a primary reason for death. The pathogenesis of CVD is complicated, including glucose or lipid metabolism disorders, endothelial dysfunction, oxidative stress, and inflammation response. Till now, atherosclerosis, myocardial ischemia, abnormally elevated blood pressure, and thrombosis are recognized as the main risk factors for inducing CVD (Benjamin et al., 2019). Plant polysaccharides from natural sources play a cardiovascular protective effect by improving these series of risk factors.

### The Effect of Plant Polysaccharides on Hypertension

**FIGURE 1:**
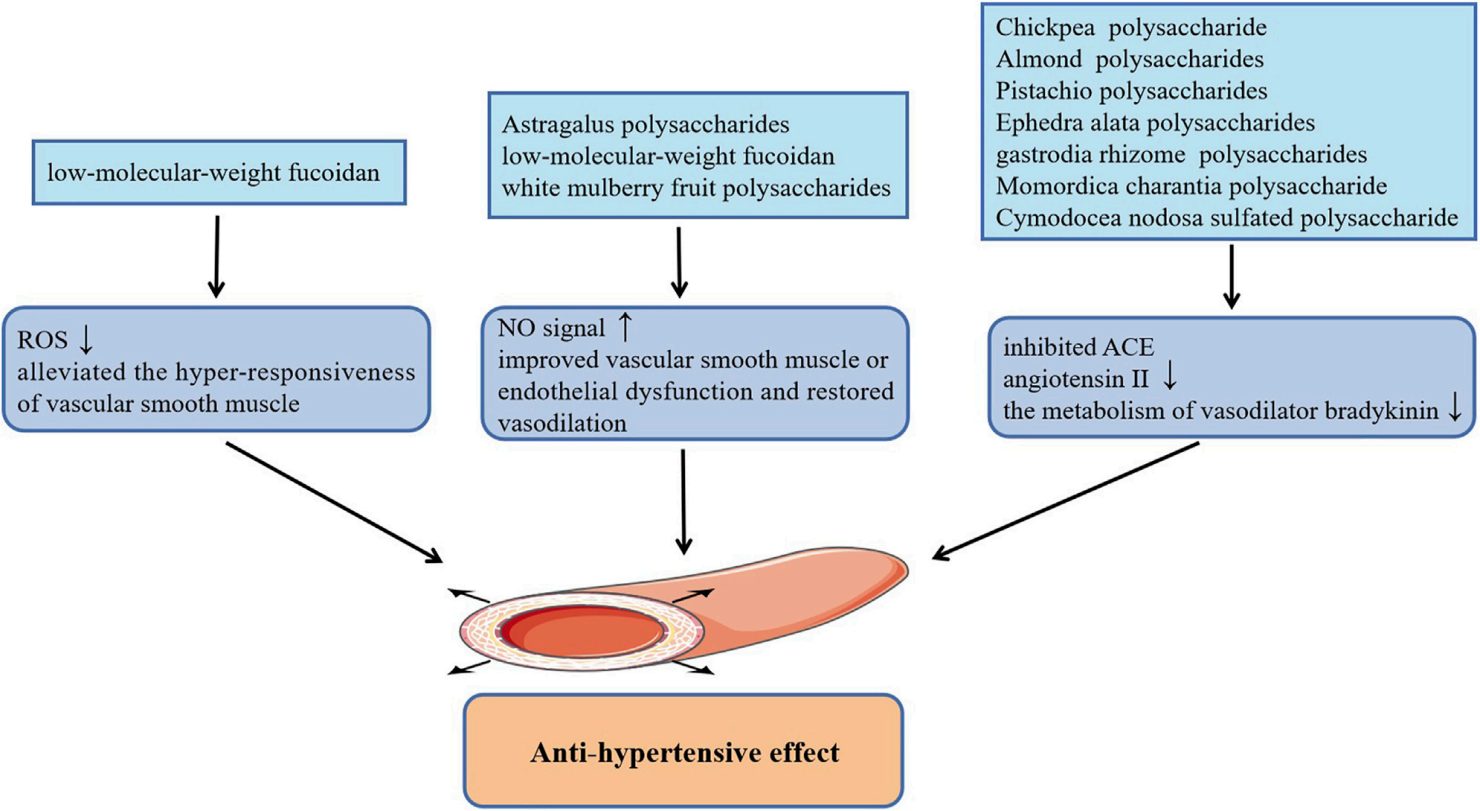
Plant polysaccharides with anti-hypertension activities.

Hypertension characterized by an uncontrolled increase in blood pressure leads to arteriosclerosis and myocardial injury, which has been regarded as one of the major factors to induce a series of refractory CVDs including coronary heart disease, cerebrovascular disease (stroke) and heart failure (Huang et al., 2013). The occurrence and development of hypertension is related to quite a few factors, among them, the dysfunction of endothelial and vascular smooth muscle is one of the primary causes of hypertension. Previous studies have shown that administration of low-molecular-weight fucoidan (LMWF) extracted from brown algae promoted the phosphorylation of endothelial nitric oxide synthase (eNOS) at Ser1177 and up-regulated the eNOs/NO signal of vascular endothelial cells, which significantly improved the vasodilation disorder induced by endothelial dysfunction and robustly reduced basal hypertension in Goto-Kakizaki type 2 diabetic rats (Cui et al., 2014). Additionally, the subsequent findings by the research group suggested that LMWF also alleviated the hyper-responsiveness of vascular smooth muscle caused by diabetes and effectively improved diabetes induced hypertension. The effect of anti-vascular smooth muscle hyper-responsiveness of LMWF is mainly achieved by restoring the activity of antioxidant enzymes to inhibit the production of ROS, and inhibiting COX-2 to reduce the level of vasoconstrictor TXA2 in vascular smooth muscle (Liang et al., 2016). Consistently, mean arterial blood pressure in both normal blood pressure rats and hypertensive rats were appreciably lowered by white mulberry fruit polysaccharides, which is also related to the increase of the release of NO in vascular endothelial cells (Wang et al., 2019). In this study, the production of NO may be related to the activation of intracellular Ca^2+^ signaling and PI3K/AKT signaling pathway. In addition, Astragalus polysaccharides treatment also reduced the mean pulmonary artery pressure in rats with monocrotaline-induced pulmonary arterial hypertension by activating eNOS/NO signaling pathway (Yuan et al., 2017). Interestingly, several studies have demonstrated that plant polysaccharides can also reduce high blood pressure by inhibiting angiotension-converting enzyme (ACE), including acidic polysaccharides from gastrodia rhizome (Lee et al., 2012), Chickpea water-soluble polysaccharide (Mokni Ghribi et al., 2015), Cymodocea nodosa sulfated polysaccharide (Kolsi et al., 2016), Momordica charantia polysaccharide (Tan and Gan, 2016), as well as water-soluble polysaccharides from Ephedra alata (Soua et al., 2020) and Almond and Pistachio (Sila et al., 2014). They can not only alleviate vasoconstriction by inhibiting the formation of angiotensin II, but also reduce metabolism of vasodilator bradykinin through the inhibition of kininase II, thereby dilating blood vessels and lowering blood pressure. Figure 1 summarizes plant polysaccharides with anti-hypertension activities.

### The Effect of Plant Polysaccharides on Atherosclerosis

Atherosclerosis (AS) is a lipid-driven vascular inflammatory disease accompanied by gradually formation of vascular occlusive plaques and thrombus at the lesion site, which in turn induces CVDs including myocardial and cerebral infraction. Polysaccharides from Nitraria retusa fruits (Rjeibi et al., 2019) improved the atherosclerosis index (AI) of hyperlipidemia mice induced by TritonX-100 by reducing the level of serum triglycerides (TG), total cholesterol (TC), low-density lipoprotein cholesterol (LDL-C), and increasing the level of high-density lipoprotein cholesterol (HDL-C). Treatment with Gastrodia rhizomes crude and acidic polysaccharides (Kim et al., 2012) also markedly reduced the content of serum TC, LDL-C and AI of high-fat diet rats, but had no effect on serum TG and HDL-C levels. Furthermore, Enteromorpha prolifera polysaccharide (Tang et al., 2013; Guo et al., 2021), polysaccharides from Porphyra yezoensis (Qian et al., 2014) also reversed abnormal serum lipid concentrations in rats or hamsters with high-fat feeding, which is beneficial to alleviate atherogenesis. Fan et al. (Fan et al., 2013) found that the effect of Okra polysaccharide in lowering the serum lipid contents of obese mice was related to the regulation of the expression of lipid metabolism-related genes. Likewise, administration of polysaccharides from Rosae laevigatae fruits (Yu et al., 2013; Zhang et al., 2020), the sulfated polysaccharide from Ulva pertusa (Qi and Sheng, 2015; Li et al., 2020) and Ophiopogon polysaccharide (Wang et al., 2017) decreased blood lipids also by affecting the expression of these genes. Surprisingly, the Ophiopogon polysaccharide (Shi et al., 2016) also lowered the blood lipid level of hyperlipidemia mice by combining with the cholesterol metabolite bile acid then promoting the excretion of cholesterol through feces. Cyclocarya paliurus polysaccharide improved the blood lipid levels of hyperlipidemia rats by up-regulating the level of lipoprotein lipase, hormone-sensitive lipase as well as adipose triglyceride lipase, which promote lipid metabolism by down-regulating the level of acetyl-CoA carboxylase, fatty acid synthase as well as hydroxy methylglutaryl coenzyme A reductase (HMG-CoA) involved in lipid synthesis (Yang et al., 2016; Hu et al., 2017). Yang et al. also reported that Cyclocarya paliurus polysaccharide can regulate the expression of lipid metabolism enzymes by affecting the methylation level of related genes, thereby reducing blood lipids (Yang et al., 2019; Yang et al., 2021). Besides, fucoidan not only promoted lipid metabolism by regulating the expression of cholesterol metabolism-related genes, but inhibited the expression of aortic *α*-smooth muscle actin (*α*-SMA), CD11b and vascular endothelial growth factor (VEGF), fibroblast growth factor-2 (FGF-2), P-SAPK as well as inflammatory cytokines, which alleviated atherosclerotic lesions in apolipoprotein E-deficient (apoE-/-) mice with high fat diet (Xu et al., 2019; Yin et al., 2019).

**FIGURE 2:**
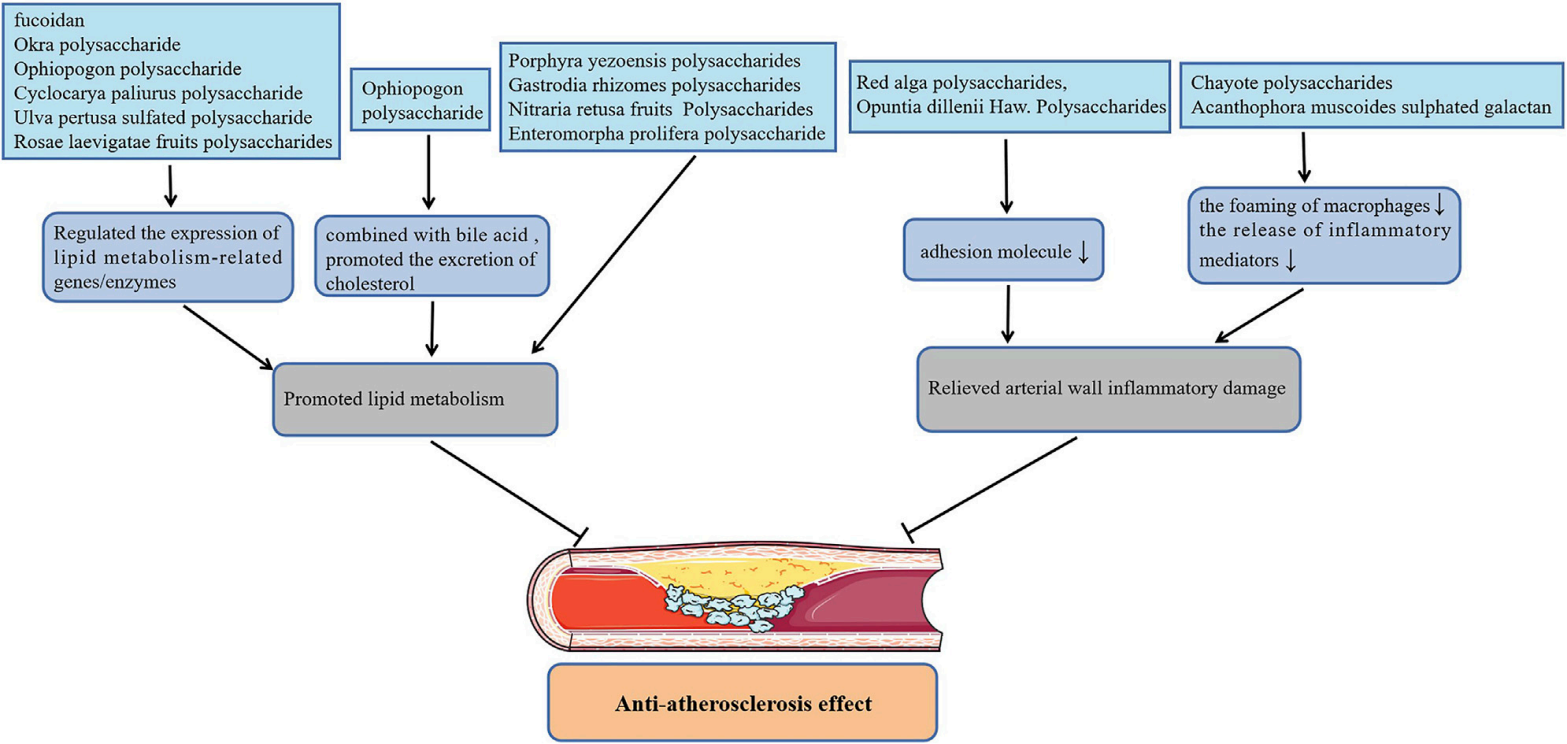
Plant polysaccharides with anti-atherosclerosis actions.

On the other hand, in atherosclerosis progression, macrophages can not only release inflammatory mediators to promote inflammatory response in the site of lesion, but excessively ingest lipids to transform into foam cells that are one of the components of atherosclerotic plaque. Remarkably, the administration of sulphated galactan isolated from the Acanthophora muscoides decreased the content of macrophages and tissue factor in the atherosclerotic plaques of apoE-/- mice with high-cholesterol diet by directly interferes with the chemotactic function of macrophages (GomesQuindere et al., 2015). In cholesterol crystals-pretreated macrophage-like THP-1 cells, treatment with Chayote polysaccharides reduced intracellular lipids levels by up-regulating the expression of liver X receptor alpha (LXR*α*), and also inhibited the activation of inflammasome NLRP3 (Castro-Alves et al., 2019). Additionally, Red alga polysaccharides inhibited the activation of NF-κB and the up-regulation of intercellular vascular cell adhesion molecule-1 (VCAM-1) as well as adhesion molecule-1 (ICAM-1) in human coronary artery endothelial cells (HCAECs) induced by angiotensin II (Hamias et al., 2018) or TNF-*α* (Levy-Ontman et al., 2017), which is helpful for alleviating inflammatory atherosclerosis progression. In addition, Opuntia dillenii Haw. Polysaccharides (Zhao et al., 2012) improved the aortic injury of hyperlipidemia rats by inhibiting the expression of VCAM-1 in the vascular endothelial and smooth muscle cells, which alleviated the process of AS. Figure 2 shows plant polysaccharides with anti-atherosclerosis actions.

### The Effect of Plant Polysaccharides on Thrombus

Thrombus is a blood clot formed by the aggregation of insoluble fibrin, activated platelets, and other cells on the internal surface of blood vessels at the site of injury, including arterial thrombosis and venous thrombosis (Chan and Weitz, 2019). Several plant polysaccharides have the biological activity of anticoagulant and inhibiting platelet aggregation, which could effectively depress the thrombosis. Guar gum hydrolysate delayed the time to arterial blood flow decreases to zero, which is beneficial to depress arterial thrombosis induced by Fecl_3_ in hamster with high-fat diet (Kuo et al., 2009). Consistently, chemically sulfated guar gum exhibited anticoagulant and antithrombotic effects in rats (de Oliveira Barddal et al., 2020). Similarly, sodium alginate sulfates inactivated *α*-thrombin and coagulation factor Xa through the interaction between negative charges in the sulfate groups and the positively charges of anti-thrombin amino acid residues, exerting anticoagulant effect (Fan et al., 2011). Differently, sulfated Citrus pectin fractions inhibited coagulation factor Xa and platelet aggregation by directly inhibiting *α*-thrombin, which attenuated venous thrombosis in rats (Cipriani et al., 2009). Additionally, sulfated rhamnan from Monostroma angicava (Liu D. et al., 2018), sulfated Pumpkin polysaccharide (Liang et al., 2018), sulfated Ginger polysaccharide (Wang et al., 2020), sulfated polysaccharides from Codium dwarkense børgesen (Golakiya et al., 2017), other sulfated polysaccharides extracted from seaweeds (Glauser et al., 2013; Chagas et al., 2020), as well as tea polysaccharides from Camellia sinensis (Cai et al., 2013) have been reported to have anticoagulant effects. On the other hand, Caesalpinia ferrea polysaccharides (de Araujo et al., 2021), polysaccharides of Geoffroea spinosa (Souza et al., 2015) and Lycium barbarum L. leaves polysaccharides (Lin et al., 2019) not only have anticoagulant activity but inhibit platelet aggregation, which exhibit depression effects on the formation of thrombus.

### The Effect of Plant Polysaccharides on Myocardial Ischemia and Myocardial Ischemia-Reperfusion Injury

**FIGURE 3:**
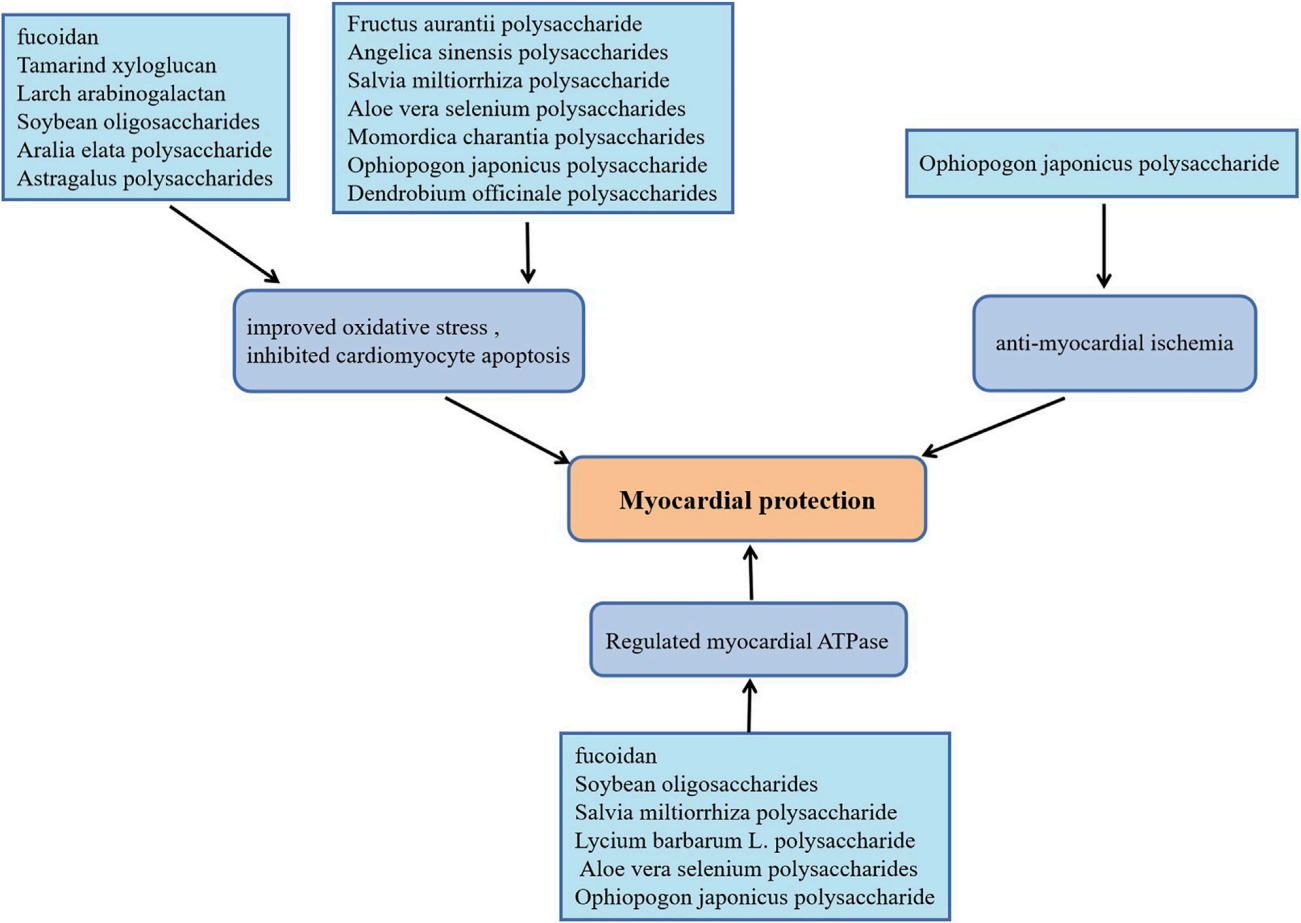
Plant polysaccharides with myocardial protective effects.

In recent years, persistent myocardial ischemia has becoming the primary cause of myocardial infarction (Thomes et al., 2010). As a classical approach, ischemia reperfusion could effectively restore the blood supply of ischemic myocardium, however, the production of a large amount of reactive oxygen species (ROS) and the infiltration of inflammatory cells caused by ischemia-reperfusion can also cause irreversible damage to the heart tissue (Hou et al., 2017). The biological activities of plant polysaccharides including anti-oxidant stress, anti-apoptosis, and anti-myocardial ischemia are beneficial to slow the progression of ischemic heart disease. Dendrobium officinale polysaccharides supplementation elevated serum SOD levels, up-regulated the expression of meis1, inhibited cardiomyocyte apoptosis, which significantly improved myocardial ischemic injury induced by coronary artery ligation in mice (Dou et al., 2016). Anti-oxidant and anti-apoptosis effects of Dendrobium officinale polysaccharide on cardiomyocytes were discovered using H9C2 cells damage model induced by H_2_O_2_ (Zhao et al., 2017). Ophiopogon japonicus polysaccharide promoted angiogenesis in myocardial ischemic tissue by activating SPHK/S1P/bFGF/AKT/ERK and eNOS/NO signaling pathways, which decreased the myocardial infarct size in rats with acute myocardial ischemia (Wang et al., 2012). It also increased endogenous antioxidants contents, Na^+^-K^+^-ATPase and Ca^2+^-Mg^2+^-ATPase activities in rats with isoproterenol (ISO)-induced myocardial ischemia (Fan et al., 2020). Momordica charantia polysaccharides protected rats against ISO-induced cardiomyocytes damage attributed to the depression of NF-κB, the increase of myocardial antioxidants levels and the decrease of pro-inflammatory factors (Raish, 2017). For rats with myocardial injury caused by cardiac ischemia or I/R, fucoidan plays a cardioprotective effect by improving oxidative stress, reducing the release of inflammatory factors and normalizing the Na^+^-K^+^-ATPase and Ca^2+^-Mg^2+^-ATPase levels (Li et al., 2011; Krishnamurthy et al., 2012). Notably, in cardiac I/R injury rats, Tamarind xyloglucan inhibited MAPK/bax/caspase-3 apoptosis cascade by up-regulated the expression of fatty acid-binding protein (Lim and Lee, 2017), while Larch arabinogalactan depressed the cardiomyocytes apoptosis by inhibiting gelsolin/MAPK p38 and gelsolin/HIF-1*α* signals, which effectively alleviated myocardial damage (Lim, 2017). Moreover, Astragalus polysaccharides (Liu X. et al., 2018), Angelica sinensis polysaccharides (Zhang et al., 2010), Aralia elata polysaccharide (Zhang et al., 2013), Aloe vera selenium polysaccharides (Yang et al., 2017), Salvia miltiorrhiza polysaccharide (Song et al., 2013; Geng et al., 2015) as well as Soybean oligosaccharides (Zhang et al., 2015) have been reported to the effects of anti-oxidation and reduce myocardial cell apoptosis in cardiac I/R model rats. More strikingly, Aloe vera selenium polysaccharides, Salvia miltiorrhiza polysaccharide and Soybean oligosaccharides also elevated the activities of Na^+^-K^+^-ATPase and Ca^2+^-Mg^2+^-ATPase, which is consistent with the effect of Lycium barbarum L. polysaccharide on cardiomyocytes in cardiac I/R rats (Hou et al., 2017). In the experiment of hypoxia-reoxygenation treatment of H9C2 cells, Fructus aurantii polysaccharide inhibited bax/caspase-mediated cells apoptosis and promoted the antioxidant effect mediated by Nrf2/HO-1 signal by activating the PI3K/AKT signaling pathway (Shu et al., 2020). Yang et al. also proved that Fructus aurantii polysaccharide has a protective effect on ISO-induced myocardial ischemia injury in rats by exerting antioxidant and anti-apoptotic effects (Yang et al., 2020). Figure 3 exhibits plant polysaccharides with myocardial protective effects.

## Discussion

In recent years, with the rise of botanical medicine, the active ingredients in traditional herbs have gradually attracted people’s attention. As one of the main active ingredients in most plant extracts, polysaccharides are widely used in research on the treatment of cardiovascular diseases. Nevertheless, most of the reports focus on the extraction, isolation, physical, and chemical properties of plant polysaccharides, but the pharmacological research of plant polysaccharides is relatively simple. We believe that the exact target of plant polysaccharides *in vivo*, and the cardiovascular protective mechanism at the molecular level need to be studied in depth in the future. On the other hand, although we generally accepted that plant polysaccharides had low toxicity or side effects, the structural uncertainty of plant polysaccharide monomers and individual differences might still lead to serious adverse events, so that the identification of plant polysaccharide molecular structure and adverse reactions clarification are necessary.
